# Supplementary material for: Resolving the full spectrum of human genome variation using Linked-Reads
Source: Genome Res. 2019 Apr;29(4):635–45. doi: 10.1101/gr.234443.118 (PMC6442396; doi:10.1101/gr.234443.118)
Supplement: Supplemental Material [file supp_gr.234443.118_Supplemental_longranger-2.2.zip › longranger-2.2.tar.bz2!/lr2.2-public/lib/stan/cmdstan/stan/src/docs/talks/nyc-ml-meetup-2012/img/hamiltonian-path.pdf]

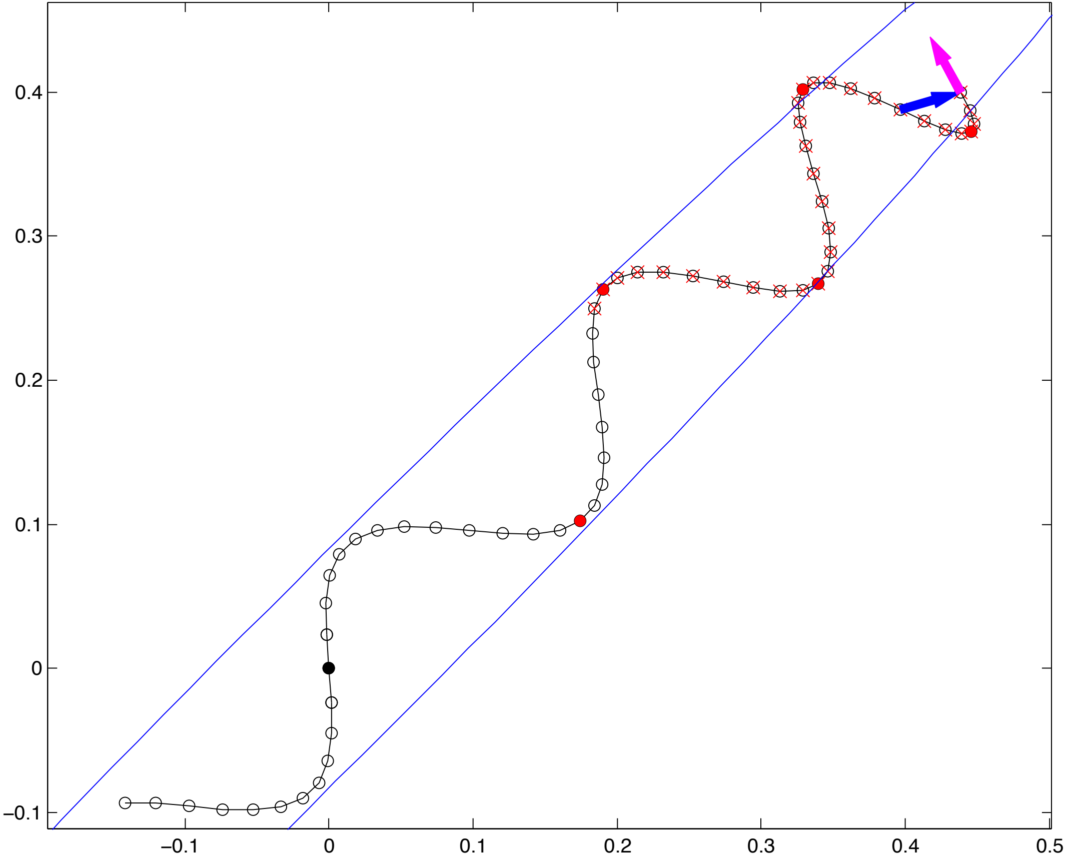

0.4
0.3
0.2
0.1
0
−0.1
−0.1
0
0.1
0.2
0.3
0.4
0.5
